# HOXA10 mediates epithelial-mesenchymal transition to promote gastric cancer metastasis partly via modulation of TGFB2/Smad/METTL3 signaling axis

**DOI:** 10.1186/s13046-021-01859-0

**Published:** 2021-02-09

**Authors:** Chenlong Song, Chongzhi Zhou

**Affiliations:** Department of General Surgery, Shanghai General Hospital, Shanghai Jiao Tong University School of Medicine, Shanghai, China

**Keywords:** HOXA10, EMT, TGFB2, Smad2/3, METTL3, Gastric cancer

## Abstract

**Background:**

Homeobox A10 (HOXA10) belongs to the HOX gene family, which plays an essential role in embryonic development and tumor progression. We previously demonstrated that HOXA10 was significantly upregulated in gastric cancer (GC) and promoted GC cell proliferation. This study was designed to investigate the role of HOXA10 in GC metastasis and explore the underlying mechanism.

**Methods:**

Immunohistochemistry (IHC) was used to evaluate the expression of HOXA10 in GC. In vitro cell migration and invasion assays as well as in vivo mice metastatic models were utilized to investigate the effects of HOXA10 on GC metastasis. GSEA, western blot, qRT-PCR and confocal immunofluorescence experiments preliminarily analyzed the relationship between HOXA10 and EMT. ChIP-qPCR, dual-luciferase reporter (DLR), co-immunoprecipitation (CoIP), colorimetric m^6^A assay and mice lung metastasis rescue models were performed to explore the mechanism by which HOXA10 accelerated the EMT process in GC.

**Results:**

In this study, we demonstrated HOXA10 was upregulated in GC patients and the difference was even more pronounced in patients with lymph node metastasis (LNM) than without. Functionally, HOXA10 promoted migration and invasion of GC cells in vitro and accelerated lung metastasis in vivo. EMT was an important mechanism responsible for HOXA10-involved metastasis. Mechanistically, we revealed HOXA10 enriched in the TGFB2 promoter region, promoted transcription, increased secretion, thus triggered the activation of TGFβ/Smad signaling with subsequent enhancement of Smad2/3 nuclear expression. Moreover, HOXA10 upregulation elevated m^6^A level and METTL3 expression in GC cells possible by regulating the TGFB2/Smad pathway. CoIP and ChIP-qPCR experiments demonstrated that Smad proteins played an important role in mediating METTL3 expression. Furthermore, we found HOXA10 and METTL3 were clinically relevant, and METTL3 was responsible for the HOXA10-mediated EMT process by performing rescue experiments with western blot and in vivo mice lung metastatic models.

**Conclusions:**

Our findings indicated the essential role of the HOXA10/TGFB2/Smad/METTL3 signaling axis in GC progression and metastasis.

**Supplementary Information:**

The online version contains supplementary material available at 10.1186/s13046-021-01859-0.

## Background

Gastric cancer (GC) is the fifth most common cancer and the third leading cause of cancer-related mortality worldwide due to aggressive properties and high metastasis potentials [1–3]. Most GC patients are first diagnosed in the metastasized stage, making the overall survival rate still low [4–7]. Therefore, it is imperative and urgent to elucidate the metastasis mechanism of GC and develop more efficient treatment strategies.

Homeobox (HOX) genes play pivotal roles in cell proliferation, apoptosis, metabolism, and migration [8]. HOXA10 is a member of the HOX gene family. Previous studies have shown that upregulated HOXA10 promoted proliferation, inhibited GC cells’ apoptosis and indicated poor prognosis for GC patients [9, 10]. However, the underlying molecular mechanism of HOXA10-mediated invasion and metastasis in GC has not been fully elucidated.

Epithelial-mesenchymal transition (EMT) is mainly executed by EMT transcription factors (EMT-TFs) and it has been widely recognized as a crucial regulator of tumor cell invasion and metastasis to distant organs [11]. Several EMT-TFs, such as Snail and Slug, could repress the E-cadherin expression, which is the fundamental event in the EMT process [12–14].

Notably, multiple studies have recognized that the transforming growth factor beta (TGFβ) signaling pathway plays a crucial role in promoting the EMT process in GC [12, 13, 15–18]. TGFβ pathway could be activated by TGF-βs (TGFβ1, TGFβ2 and TGFβ3), which are secreted by cells and have similar but not identical functions [13]. Interestingly, recent research has revealed that TGFB2 is upregulated in GC and predicted poor survival [19]. Besides, a previous study in myeloid progenitor cells showed HOXA10 could activate TGFB2 transcription and increase TGFβ2 production [20]. So far, however, the relationship between HOXA10 and TGFβ signaling in GC has not been clearly understood, which prompted us to perform further exploration.

TGF-βs could activate canonical TGFβ signaling and subsequently phosphorylate Smad2/3, which are the main intracellular effectors of the TGFβ pathway [13, 21, 22]. It is noteworthy that Alessandro Bertero et al. gave us new insights that Smad2/3 could interact with the METTL3-METTL14-WTAP complex in hESCs and hiPSCs, thus promoting N6-methyladenosine (m^6^A) modification on specific Smad2/3 transcriptional targets [23].

M^6^A is the most common mRNA modification in eukaryotes and regulates mRNA stability, splicing, translation and degradation [24, 25]. METTL3, METTL14 and WTAP are the main m^6^A “writers,” of which METTL3 is the primary catalytic subunit and dysregulated METTL3 could result in changes of the total m^6^A methylation [26–28].

Recent studies have shown elevated METTL3 promotes EMT in cancers [29–31], and several EMT-TFs (such as Snail and Slug) are key molecules of EMT [12, 13]. It should also be noted that Lin et al. demonstrated m^6^A modification on Snail, which acted as a Smad2/3 target, promoted its translation in cancer cells and accelerated the EMT progression [32, 33]. Therefore, we mainly focused on the relationship between HOXA10 and METTL3 to explore whether HOXA10 might promote the EMT process and accelerate GC metastasis via activating TGFβ/Smad signaling and regulating m^6^A modification.

In this study, we demonstrated that HOXA10 promoted metastasis of GC by inducing EMT. The relevant mechanism could be explained as follows: elevated HOXA10 enhanced the transcription and secretion of TGFB2, activated TGFβ/Smad signaling, promoted the interaction of Smad2/3 with METTL3 in the nucleus, and upregulated the expression of Snail and Slug through m^6^A modification.

## Materials and methods

### Patients and specimens

A total of 127 paired GC tissue specimens collected for immunohistochemistry and other 80 paired GC tissue samples selected for qRT-PCR were obtained from Shanghai General Hospital for this research. This study was approved by the Ethics Committee of Shanghai General Hospital and all patients provided informed consent.

### Immunohistochemistry (IHC) staining

The immunohistochemistry (IHC) analysis was performed using a Dako K5007 kit (Dako, Denmark) according to the manual instructions. The staining intensity was scored as (0 = negative, 1 = weak, 2 = moderate, 3 = strong) according to the expression level and the staining area was scored as (0 = 0%, 1 = 1–25%, 2 = 26–50%, 3 = 51–100%) based on the percentage of positive cells. The immunostaining score (IS) was calculated by adding the score of staining intensity and area according to the method of Yuan et al. [34]. The positive specimen for HOXA10 and E-cadherin individually was defined as an IS≥3, while negative staining with an IS < 3. The primary antibodies used in immunohistochemistry included: HOXA10 (1:100, Santa Cruz), E-cadherin (1:400, CST), N-cadherin (1:125, CST), Vimentin (1:200, CST), METTL3 (1:500, Abcam).

### Cell culture, lentivirus construction and transfection

Stable HOXA10 knockdown (BGC823-shHOXA10) or overexpression (AGS-HOXA10) GC cells have been successfully established [9]. The cells were divided into the categories below: AGS-Mock, without any treatment; AGS-Ctrl, infected with the lentivirus containing the control fragment; AGS-HOXA10, infected with the lentivirus containing the HOXA10 fragment; BGC823-Mock, without any treatment; BGC823-Ctrl, infected with the control Lenti-shRNA;BGC823-shHOXA10, infected with the HOXA10 Lenti-shRNA. The cells were cultured using an RPMI1640 medium containing 10% fetal bovine serum (Gibco, USA).

The lentivirus containing METTL3 shRNA (sh-METTL3) or negative control shRNA (sh-NC) and the METTL3 overexpression lentivirus (METTL3) or control vector lentivirus (Vector) were obtained from Obio Technology (Shanghai). Stable METTL3 overexpression or knockdown in previously selected HOXA10 differential expressing GC cells was achieved by transfecting the lentivirus.

### Cell scratch wound-healing assay

The cells were plated in six-well plates and cultured until full confluence. Then suitable micropipette tips were used to generate uniformed scratches from the center of each well. After washed with phosphate-buffered saline (PBS), the cells were then cultured in fetal bovine serum (FBS)-free medium and taken photos at 0 h, 48 h. The cell migration was measured by comparing the gap change with the initial gap at 0 h. The experiment was performed independently in triplicate.

### Cell transwell migration and invasion assays

The transwell 24-well chambers with 8.0 μm pore size (Corning, USA) were used for cell migration and invasion assays. The cells dispersed in 200 μL serum-free medium (2 × 10^4^/ well) were seeded into the upper chamber, and 600 μL medium containing 10% FBS was added to the lower section. The upper compartment was coated with Matrigel (BD Bioscience, USA) for invasion assay or without for migration assay. The cells migrated or invaded to the lower chamber at time point 24 h or 36 h respectively were stained with 0.1% crystal violet and then counted. Each experiment was conducted independently in triplicate.

### Nude mice metastasis models

4-week male athymic BALB/c nude mice were used to establish in vivo metastasis mice models. For the lung metastasis models, 5 × 10^6^ different stable transfected cells were injected through the mice tail vein (*n* = 5 for each group). Luciferase imaging was performed to monitor metastatic progression with an IVIS-100 system (Caliper Life Sciences, USA) after injection of D-Luciferin potassium salt (Synchem, Germany) intraperitoneally.

For the liver metastasis models, 5 × 10^6^ cells (BGC823-Ctrl or BGC823-shHOXA10) resuspended in sterile PBS were injected into the spleen of nude mice.

All the nude mice were killed after 6 weeks, and all the lungs and livers were collected for HE staining or immunohistochemistry analysis. All the animal studies were performed strictly following the Animal Care Guidelines approved by the Animal Care Committee of Shanghai General Hospital.

### RNA-sequencing and GSEA

For RNA-Sequencing, total RNA separated from BGC823-Ctrl and BGC-shHOXA10 were extracted using Trizol reagent (Takara, Japan). Majorbio Bio-pharm Technology Corporation performed the construction of the transcriptome libraries and Illumina sequencing, and each group contained three biological replicates. Gene Set Enrichment Analysis (GSEA) was conducted using the GSEA software downloaded from the Broad Institute website (https://www.gsea-msigdb.org/gsea/index.jsp).

### Western blot analysis

Total cellular proteins or nuclear proteins were separately extracted using RIPA buffer (Beyotime Biotechnology, China) or Minute™ Cytoplasmic and Nuclear Extraction Kit for Cells (Invent Biotechnologies, USA). In brief, the protein samples were separated using SDS-PAGE gel (7.5–12%) electrophoresis and then transferred to PVDF membranes. After blockade in 5% skimmed milk, the membranes were incubated with primary antibodies at 4 °C overnight followed by incubation of specific second antibodies for 1 h at room temperature. The target proteins were then detected using ECL reagent kits (Millipore, USA). The primary antibodies used in western blot were listed in Table 1.

**Table 1:** Primary antibodies for western blot

Antibody	Concentration	Specificity	Company
HOXA10	1:1000	Mouse	Santa Cruz
E-cadherin	1:1000	Rabbit	Cell Signaling Technology
N-cadherin	1:1000	Rabbit	Cell Signaling Technology
Vimentin	1:1000	Rabbit	Cell Signaling Technology
Snail	1:1000	Rabbit	Cell Signaling Technology
Slug	1:1000	Rabbit	Cell Signaling Technology
TGFB1	1:2000	Rabbit	Proteintech
TGFB2	1:1000	Mouse	Abcam
TGFB3	1:500	Rabbit	Proteintech
GAPDH	1:5000	Mouse	Proteintech
Smad2	1:1000	Rabbit	Cell Signaling Technology
P-Smad2	1:1000	Rabbit	Cell Signaling Technology
Smad3	1:1000	Rabbit	Cell Signaling Technology
P-Smad3	1:1000	Rabbit	Cell Signaling Technology
Smad2/3	1:1000	Rabbit	Cell Signaling Technology
Histone H3	1:2000	Rabbit	Cell Signaling Technology
P-Smad2/3	1:1000	Rabbit	Cell Signaling Technology
METTL3	1:1000	Rabbit	Abcam

### Quantitative real-time PCR (qRT-PCR)

For qRT-PCR, total RNA extraction from all cell lines and GC tissues was performed using Eastep® Super Total RNA Extraction Kit (Promega, USA) according to the instructions. RNA was reversely transcribed to cDNA using PrimeScript™ RT Master Mix (Takara, Japan). qRT-PCR reactions were carried out using the Hieff™ qPCR SYBR Green Master Mix (Yeasen, China).

The following primer sequences used for qRT-PCR were listed in Table 2:

**Table 2:** Sequences of primers for qRT-PCR

Gene	Forward primer	Reverse primer
HOXA10	TCACGGCAAAGAGTGGTC	AGTTTCATCCTGCGGTTCTG
E-cadherin	AGGCCAAGCAGCAGTACATT	CATTCACATCCAGCACATCC
N-cadherin	TTTGAGGGCACATGCAGTAG	ACTGTCCCATTCCAAACCTG
Vimentin	CGAAACTTCTCAGCATCACG	GCAGAAAGGCACTTGAAAGC
Snail	CCTCGCTGCCAATGCTCATCTG	GCTCTGCCACCCTGGGACTC
Slug	TCTTCACTCCGAAGCCAAAT	TCTGTGGGTGTGTGTGTGTG
TGFB1	CTGTACATTGACTTCCGCAAG	TGTCCAGGCTCCAAATGTAG
TGFB2	GCAAAGTTGTGAAAACAAGAGC	ATCCCAGGTTCCTGTCTTTATG
TGFB3	CTGTTGAGAAGAGAGTCCAACT	GATTTCCATCACCTCGTGAATG
METTL3	CCAGCACAGCTTCAGCAGTTCC	GCGTGGAGATGGCAAGACAGATG
GAPDH	GGGAAGGTGAAGGTCGGAGT	GGGGTCATTGATGGCAACA

HOXA10; E-cadherin; N-cadherin; Vimentin; Snail; Slug; TGFB1; TGFB2; TGFB3; METTL3; GAPDH.

The relative mRNA quantification was calculated using the 2^−ΔΔCt^ method and normalized by GAPDH. All the experiments were repeated in triplicate.

### Confocal immunofluorescence staining

Cells were seeded on confocal dishes (Cellvis, USA) for 24 h before fixed with paraformaldehyde and permeabilized by Triton X-100. After blocking with 5% normal donkey serum, the cells were incubated at 4 °C overnight with primary antibodies specific against E-cadherin (1:200, CST), N-cadherin (1:200, CST), Vimentin (1:100, CST), Smad2/3 (1:400, CST), followed by fluorescence-conjugated secondary antibodies for 1 h at room temperature. The cells were then counterstained with DAPI (Sigma, Germany) and were photographed using a confocal laser scanning microscope (Leica, Germany).

### Chromatin immunoprecipitation (ChIP)

ChIP assay was performed using the SimpleChIP® Plus Enzymatic Chromatin IP Kit (Magnetic Beads) (CST, USA) following the same protocol as previously described [9]. For the ChIP-qPCR assay, primers explicitly designed to target the TGFB2 or METTL3 promoter region were listed in Table 3. The products of ChIP-qPCR were qualitatively analyzed by 3% agarose gel electrophoresis.

**Table 3:** Sequences of primers for ChIP-qPCR

ChIP-qPCR primer	Forward primer	Reverse primer
TGFB2-P1	GGTGAAGAAGAAAGCCATACAAGAAGT	CCTCTGTTTGTGAACTGCTGGAAA
TGFB2-P2	AGGGAAGGTGGAACAGTGGTAAG	TCTTTCCATAGCATTAATCCAGGAAGC
TGFB2-P3	CTTTGTACCTCAGTCTCCTCATCTGC	GGGGCCTAGGTTATTAGTAGAGCAC
TGFB2-P4	AGGTGCATCAGTGTCTGTACCA	TGAGAGAAGATGCTGCTGTGGA
METTL3-P1	TGCCCATGCTACCTCATGCT	TGACTGGCATGGCTCCTGTT
METTL3-P2	ACACCAGCCTGGGCAGTAAG	ATCTGCCCCTACGATCTGCC
METTL3-P3	AGGAACTAGTGATCCTTCATCT	CTCCTCACCTCGTGATCC

### Dual-luciferase reporter gene assay

For the dual-luciferase reporter assay, pGL3-TGFB2 (TGFB2-WT) and pGL3-mutated TGFB2 (TGFB2-mut) plasmids were constructed and synthesized by Zorin (Zorin, China). Besides, Flag-tagged HOXA10 overexpressing plasmid and control plasmid were reconstructed and acquired from GeneChem (GeneChem, China). 293T, AGS and BGC823 cells were co-transfected with the luciferase reporter plasmid and other indicated expression constructs using Lipofectamine 2000 (Invitrogen, USA).

The luciferase assays were performed after transfection for 48 h by using a Dual-Luciferase Reporter Assay System kit (Promega, USA). Then the relative luciferase activity was detected and normalized to Renilla activity.

### TCA protein precipitation assay

The TCA Protein Precipitation Kit (Sangon Biotech, China) was used to separate protein pellets from cellular supernatant in different HOXA10 expression cells according to the manufacturers’ instructions. The protein extracts from TCA assays were then used for western blot detection.

### ELISA assay

TGFβ2 concentration in cellular supernatant from altered HOXA10 expression cells was quantified with a human TGFβ2 ELISA kit (MultiSciences, China) according to the manual guides. All the samples were repeated in triplicate.

### Co-immunoprecipitation assay

For each immunoprecipitation assay (IP), nuclear proteins extracted from 2 × 15 cm dishes BGC823 cells with full confluence were used according to the manufacturer’s protocol (Invent Biotechnologies, USA). The nuclear protein lysates were immunoprecipitated with specific antibodies targeting against Smad2/3 (CST, USA), METTL3 (Abcam, USA) or IgG negative control (CST, USA) at 4 °C overnight in the rotator, then added into 50 μL Protein A/G Magnetic Beads (MedChemExpress, USA) pre-cleaned with PBST to rotate for 2 h at 4 °C. Subsequently, the magnetic beads were washed 4 times with PBST buffer, and the immunoprecipitation complexes were separated from the beads for further western blot analysis.

### Colorimetric m^6^A assay

The relative N6-methyladenosine (m^6^A) levels in GC cells with different HOXA10 expression were measured via the EpiQuik™ m^6^A RNA Methylation Quantification Kit (Colorimetric) (Epigentek, USA) according to the manufacture’s guidance.

### Statistical analysis

All data were analyzed with IBM SPSS Statistics version 23.0 software (SPSS, USA). The statistical significance between HOXA10 or E-cadherin protein levels with the clinicopathologic characteristics levels was measured using the Chi-square test.

The correlation between HOXA10 and E-cadherin protein was calculated with the *Spearman* correlation coefficient test, while the correlation between HOXA10 and METTL3 mRNA expression was analyzed using *Pearson* correlation analysis.

Quantitative results for in vitro and in vivo experiments were shown as mean ± SD or box plot, and the differences were determined using the two-tailed Student’s *t-test*. For all studies, *P*-values < 0.05 indicates statistically significant differences.

## Results

### HOXA10 overexpression is associated with metastasis in GC

We previously demonstrated that HOXA10 was upregulated in GC tissues, and the Kaplan-Meier Plotter database showed higher expression of HOXA10 predicted poor prognosis in GC patients [9].

**Fig. 1:**
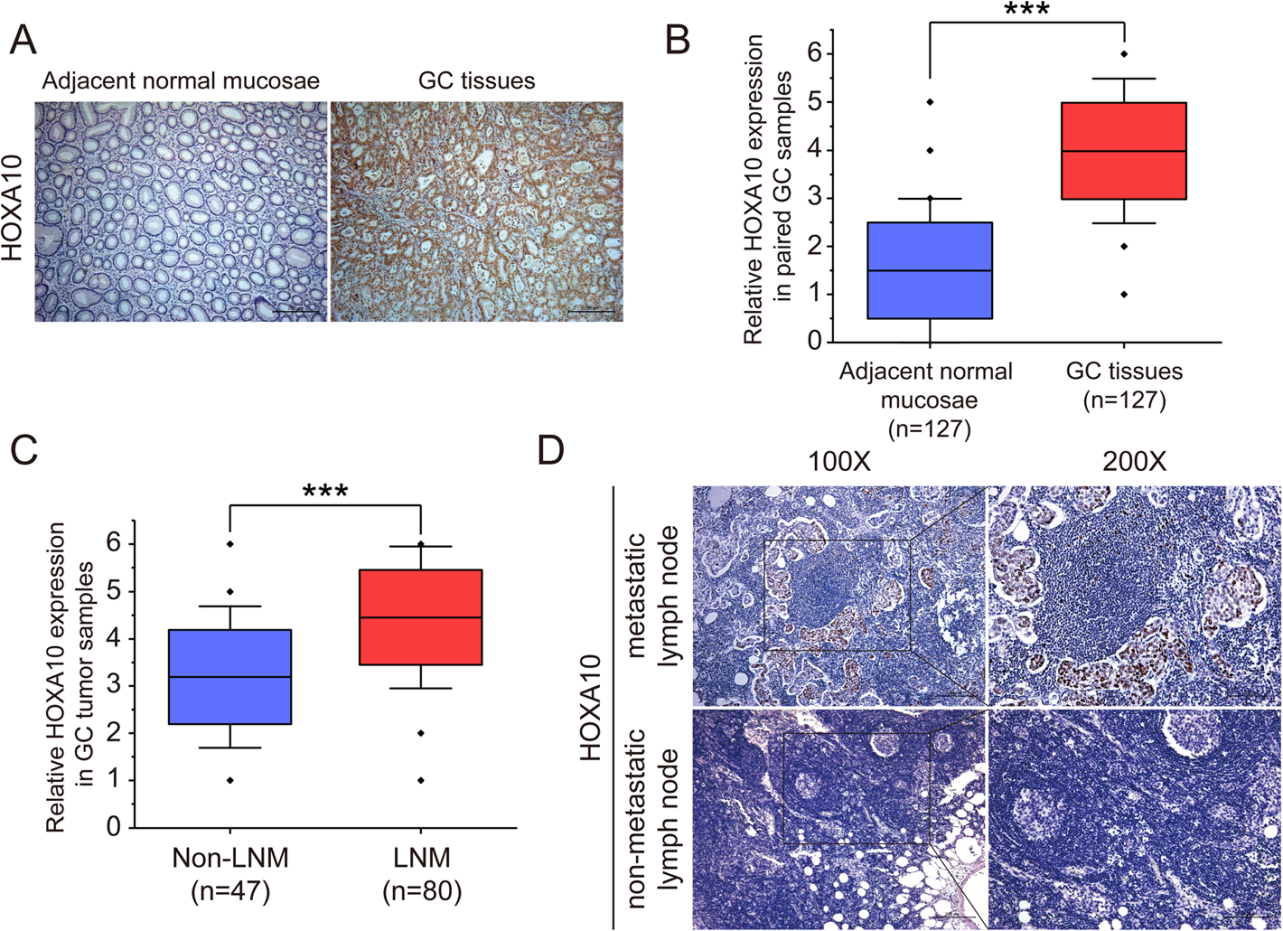
HOXA10 upregulation in GC was associated with metastasis. **a** Representative images of immunohistochemistry for HOXA10 in primary GC tumor samples and adjacent normal mucosae. **b** Immunohistochemistry analysis of HOXA10 expression levels in GC tumor samples and their paired adjacent normal mucosae. **c** Immunohistochemistry analysis of HOXA10 expression in GC tumor samples without or with LNM. **d** Representative immunohistochemical staining of HOX10 in metastatic lymph node and non-metastatic lymph node. Original magnification, 100× (200× for locally magnified images). ***P < 0.001

Herein to further investigate the role of HOXA10 in GC progression, we employed 127 pairs of GC samples to analyze the expression levels of HOXA10. The results showed that HOXA10 was significantly upregulated in the GC tumor samples than the adjacent normal mucosae (mean IS = 3.98 vs. 1.50, *P* < 0.001, Fig.1a, b). More importantly, we found HOXA10 had higher expression in GC tumor samples with LNM than those of non-LNM (mean IS = 4.45 vs. 3.19, *P* < 0.001; Table 4 and Fig. 1c). Besides, we observed that HOXA10 showed positive staining in metastatic lymph nodes (Fig. 1d). Taken together, these results suggested HOXA10 upregulation might be relevant to metastasis of GC.

**Table 4:** Relationship between clinicopathologic parameters and the expression of HOXA10, E-cadherin in gastric cancer (*n* = 127)

Characteristics	N	HOXA10			E-cadherin		
		+	–	*P-value*	+	–	*P-value*
Age				0.417			0.913
< 65	59	38	21		22	37	
≥ 65	68	39	29		26	42	
Gender				0.811			0.509
Male	72	43	29		29	43	
Female	55	34	21		19	36	
Location				0.956			0.226
Gastric fundus	14	9	5		8	6	
Gastric corpus	53	32	21		17	36	
Pylorus	60	36	24		23	37	
Tumor size				0.005			0.292
< 3 cm	38	16	22		17	21	
≥ 3 cm	89	61	28		31	58	
UICC stage				< 0.001			0.075
I + II	56	24	32		26	30	
III + IV	71	53	18		22	49	
Lymph node metastasis				< 0.001			0.002
No	47	18	29		26	21	
Yes	80	59	21		22	58	

### HOXA10 promotes GC cells migratory and invasive capacities in vitro

Given that HOXA10 was upregulated in GC tissues and was associated with metastasis, we then investigated whether HOXA10 could influence the GC cell metastatic capacity in vitro.

**Fig. 2:**
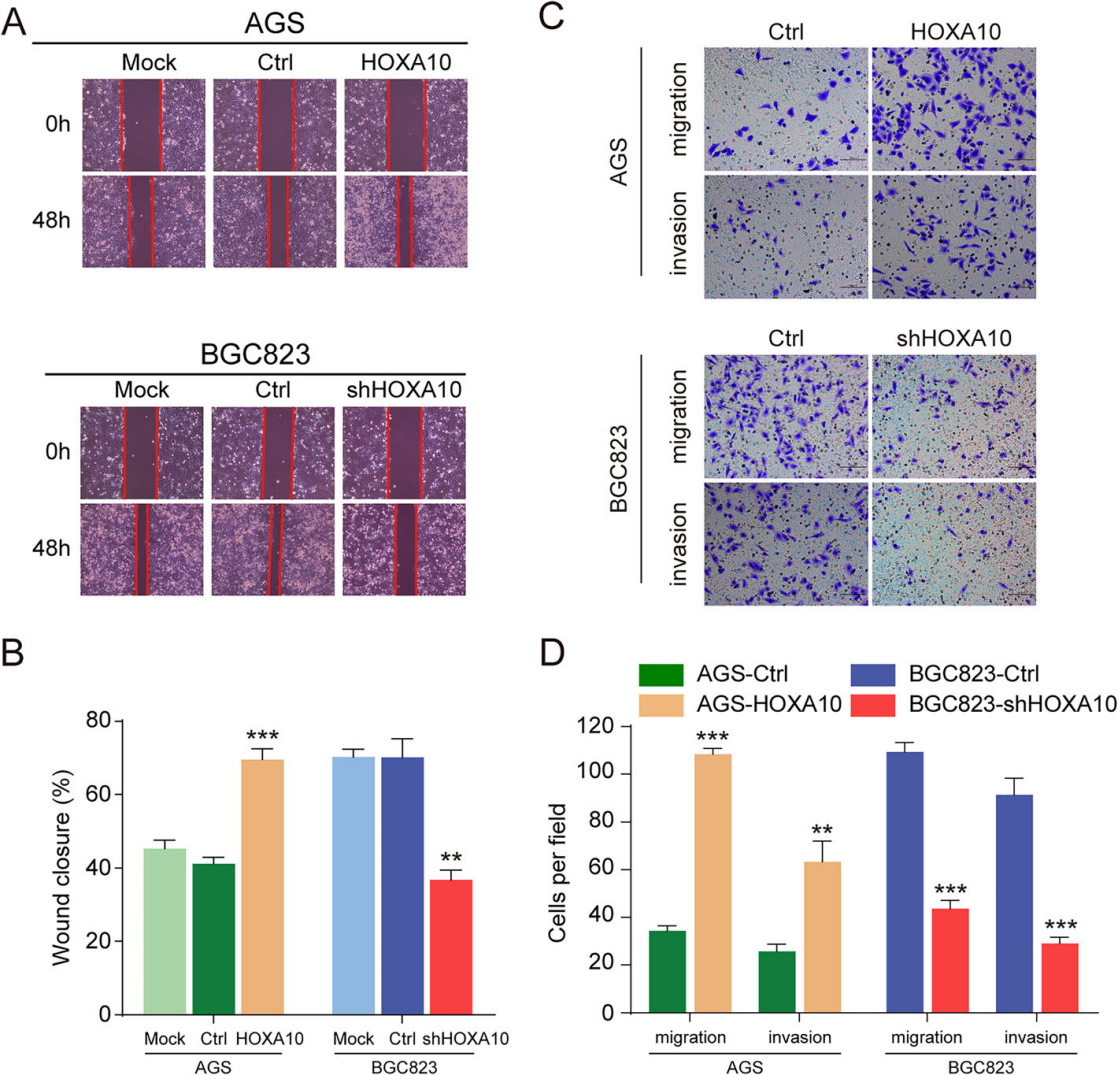
HOXA10 promoted GC cells migratory and invasive capacities in vitro. **a-b** Overexpression or knockdown of HOXA10 increased or decreased GC cells’ wound healing abilities. **c-d** Upregulation or downregulation of HOXA10 enhanced or impaired GC cells’ migration and invasion abilities. Original magnification, 100× (wound healing assay), 200× (transwell migration and invasion assays). **P < 0.01, ***P < 0.001

We have established the HOXA10 overexpressing tool cell (AGS-HOXA10) and HOXA10 knockdown tool cell (BGC823-shHOXA10) [9]. Wound-healing assay verified that AGS-HOXA10 cells closed the wound area significantly faster while BGC823-shHOXA10 cells relatively slower than respective control cells. (Fig. 2a-b). Moreover, the transwell assays indicated that overexpression of HOXA10 significantly promoted the migratory and invasive abilities of AGS cells. On the contrary, HOXA10 downregulation obviously reduced the abilities in BGC823 cells as well (Fig. 2c-d). These results suggested that HOXA10 promoted GC cells metastatic abilities in vitro.

### HOXA10 enhances GC cells metastasis in vivo

**Fig. 3:**
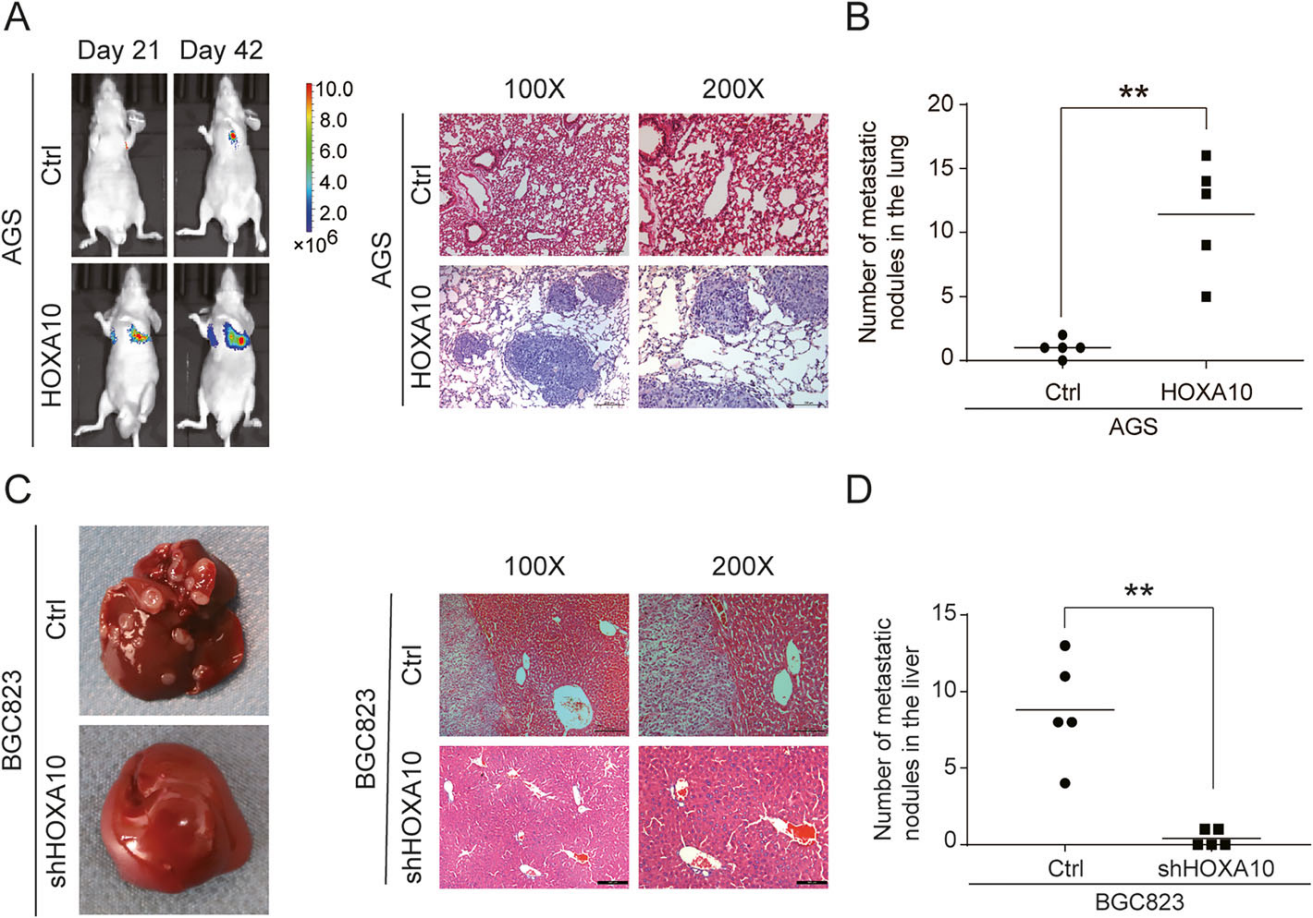
HOXA10 promoted GC cells metastatic abilities in vivo*.* **a** AGS-Ctrl and AGS-HOXA10 cells were intravenously injected into the nude mice to establish lung metastasis models. Representative pictures of luciferase imaging and H&E staining were shown. **b** The numbers of metastatic lung nodules. **c** BGC823-Ctrl and BGC823-shHOXA10 cells were injected into the spleen of nude mice to establish hepatic metastasis models. Representative images of metastatic liver nodules and H&E staining were shown. **d** The numbers of hepatic metastasis nodules. **P < 0.01

To investigate the in vivo effect of HOXA10 on GC cells metastasis, we performed two metastatic assays. After AGS-HOXA10 and control cells were injected into nude mice via the tail vein, we collected images at different time points to dynamically observe the location and enlargement of lung metastases. The result showed that HOXA10 overexpression remarkably increased the number of metastatic lung nodules (Fig. 3a-b). Also, the nude mice liver metastasis model was established via injecting modified BGC823 cells into the spleens. Notably, HOXA10 silencing obviously reduced the number of metastatic liver tumors (Fig. 3c-d). The above experiments demonstrated that HOXA10 enhanced GC cells metastasis in vivo.

### HOXA10 promotes GC cells metastasis by modulating EMT

To better understand the role of HOXA10 in GC metastasis, transcriptome sequencing analysis (RNA-Seq) was used to compare the relative changes of gene expression in BGC823-Ctrl and BGC823-shHOXA10 cells. Then Gene Set Enrichment Analysis (GSEA) demonstrated that HOXA10 knockdown was negatively correlated with EMT, which meant downregulated HOXA10 inhibited EMT pathway in GC (Fig. S1).

EMT is a crucial mechanism that leads to cancer metastasis, and the loss of E-cadherin is commonly recognized as a molecular marker in EMT progression [11]. Then qRT-PCR, Western blot and confocal immunofluorescence analysis were performed to validate further whether EMT was responsible for HOXA10 induced metastatic phenotype in GC.

**Fig. 4:**
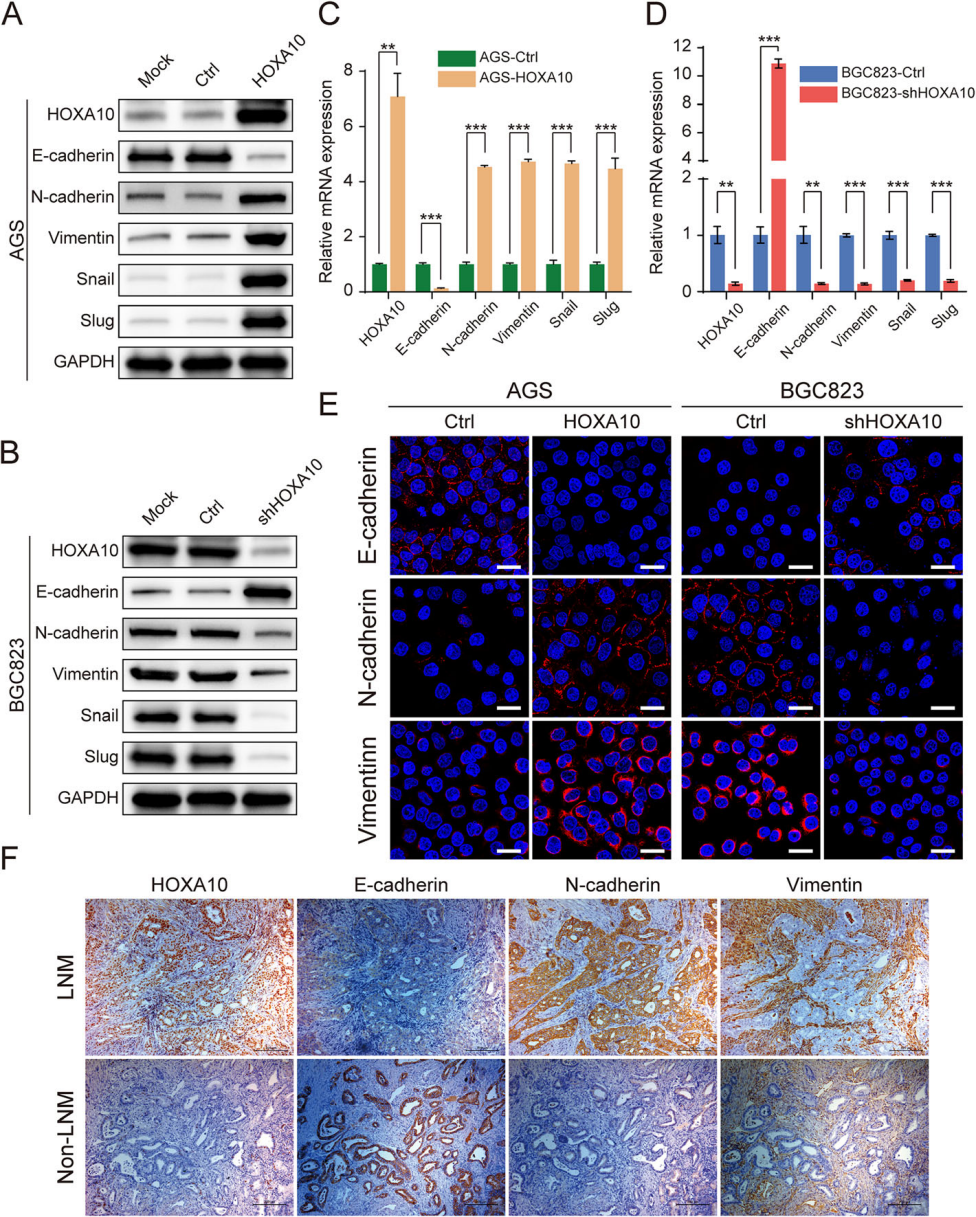
HOXA10 modulated the expression of EMT-related molecules in GC cells. **a-d** Expression of EMT-related molecules (E-cadherin, N-cadherin, Vimentin, Snail, Slug) in indicated groups of AGS and BGC823 cells were analyzed by western blot (a-b) and qRT-PCR (c-d). **e** Representative confocal Immunofluorescence staining of E-cadherin, N-cadherin and Vimentin in different GC cells with modified HOXA10 expression. **f** Representative IHC staining of HOXA10, E-cadherin, N-cadherin and Vimentin in GC cancer tissues with or without LNM. Immunofluorescence scale bars: 25 μm. IHC original magnification, 100×. **P < 0.01, ***P < 0.001

As shown in Fig. 4a, c, compared with the AGS-Ctrl cells, AGS-HOXA10 cells expressed a lower level of epithelial marker E-cadherin and a higher level of the mesenchymal markers N-cadherin, Vimentin as well as EMT-TFs Snail and Slug. In contrast, knockdown of HOXA10 in BGC823 cells elevated the expression of E-cadherin, which was accompanied by the suppression of N-cadherin, Vimentin, Snail and Slug (Fig. 4b, d). Moreover, the changes of characterized EMT markers in different HOXA10-expressing cells were further validated by confocal immunofluorescence (Fig. 4e).

Subsequently, we performed IHC in typical GC serial sections with or without LNM and found that HOXA10 expression was consistent with N-cadherin and Vimentin but seemed to be opposite to E-cadherin (Fig. 4f). Furthermore, we analyzed the expression correlation between HOXA10 and E-cadherin in 127 paired GC tumor samples and found that HOXA10 was inversely correlated with E-cadherin (r = − 0.469, *P* < 0.001; Table 5).

**Table 5:** Association between HOXA10 and E-cadherin expression in GC tumor samples

GC Samples	E-cadherin		r	*P* value
	Negative	Positive		
HOXA10 negative	17	33	−0.469	< 0.001
HOXA10 positive	62	15		

Besides, the relationships between clinicopathologic features and the expression of HOXA10 or E-cadherin in GC patients were summarized in Table 4. Interestingly, both proteins were significantly related to LNM (*P* < 0.01). In summary, these findings suggested that HOXA10 might enhance GC cells metastasis through EMT.

### HOXA10 modulates TGFβ signaling pathway possibly by regulating TGFB2 in GC

**Fig. 5:**
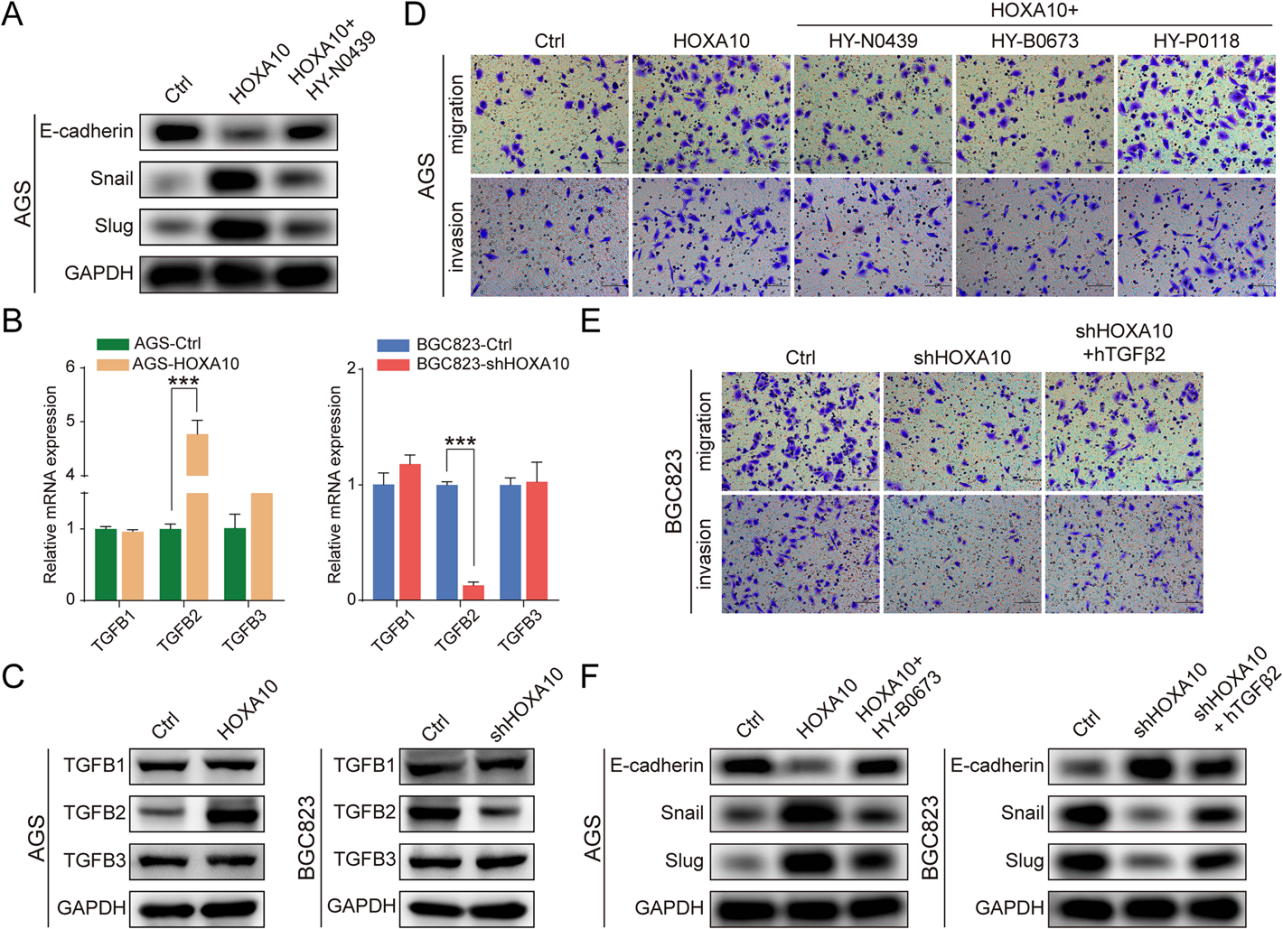
HOXA10 modulated TGFβ signaling pathway principally by regulating TGFB2 in GC. **a** The protein expression in AGS-HOXA10 cells after TGFβ inhibitor (HY-N0439) treatment. **b-c** The mRNA or protein levels of TGFB1, TGFB2 and TGFB3 in different GC cells with modified HOXA10 expression. **d** The alteration of migratory and invasive abilities in AGS-HOXA10 cells after being treated with TGFβ, TGFβ1 or TGFβ2 inhibitor. **e** The alteration of migratory and invasive abilities in BGC823-shHOXA10 cells with treatment of hTGFβ2. **f** The protein expression in AGS-HOXA10 cells after TGFβ2 inhibitor (HY-N0673) treatment or in BGC823-shHOXA10 cells after the treatment of hTGFβ2 cytokine. Original magnification, 200×. ***P < 0.001

TGFβ signaling has been recognized as essential in inducing GC EMT progression [17]. To further study whether HOXA10 promoted EMT and GC metastasis through the canonical TGFβ pathway, we first treated AGS-HOXA10 cells with TGFβ/Smad signaling inhibitor HY-N0439 (MedChemExpress, USA). As shown in Fig. 5a, HY-N0439 upregulated the expression of E-cadherin while downregulated Snail and Slug. This result suggested that HOXA10-induced EMT progress might be suppressed by blocking TGFβ/Smad signaling.

Then we investigated which isoform of TGFβ played a role in HOXA10 mediating EMT. It should be noted that qPCR and western blot results showed only TGFB2 expression, but not TGFB1 or TGFB3, was significantly increased by upregulating HOXA10 (Fig. 5b, c).

Moreover, AGS-HOXA10 cells were further processed with selective TGFβ inhibitor (HY-N0439), TGFβ2 inhibitor (HY-B0673) (MedChemExpress) or TGFβ1 inhibitor (HY-P0118) (MedChemExpress). The results showed cell migration/invasion induced by HOXA10 overexpression was significantly inhibited by 47.6%/31.9% or 48.2%/30.8% after being treated with HY-N0439 or HY-B0673, while only 13.4%/7.7% with HY-P0118 (Fig. 5d; Fig. S2). Similarly, cell migration/invasion ability was partly restored in BGC823-shHOXA10 cells after cytokine hTGFβ2 treatment (Fig. 5e; Fig. S2).

Additionally, western blot analysis demonstrated the decrease of Snail and Slug accompanied by the elevation of E-cadherin when TGFβ2 activity was inhibited using HY-B0673 in AGS-HOXA10 cells. In contrast, the opposite trend was observed in BGC823-shHOXA10 cells when stimulated with hTGFβ2 (Fig. 5f).

The above results indicated that HOXA10 was involved in modulating TGFβ signaling possibly by regulating TGFB2 expression.

### HOXA10 activates TGFB2 expression through transcriptional regulation

Interestingly, the TRANSFAC database (http://gene-regulation.com/) predicted that TGFB2 might be a target gene through transcriptional regulation by HOXA10 (Table 6).

**Table 6:** Gene bound by HOXA10 predicted by TRANSFAC

Search Term	Name	Species/Taxon
GN000009303-HOXA10	CDKN1A	Human
GN000009303-HOXA10	MAFB	Human
GN000009303-HOXA10	KLF9	Human
GN000009303-HOXA10	KAT2B	Human
GN000009303-HOXA10	ARIH2	Human
GN000009303-HOXA10	CDX4	Human
GN000009303-HOXA10	CYBB	Human
GN000009303-HOXA10	DUSP4	Human
GN000009303-HOXA10	FGF2	Human
GN000009303-HOXA10	TGFB2	Human
GN000009303-HOXA10	MYF5	Human
GN000009303-HOXA10	EMX2	Human
GN000009303-HOXA10	ITGB3	Human

**Fig. 6:**
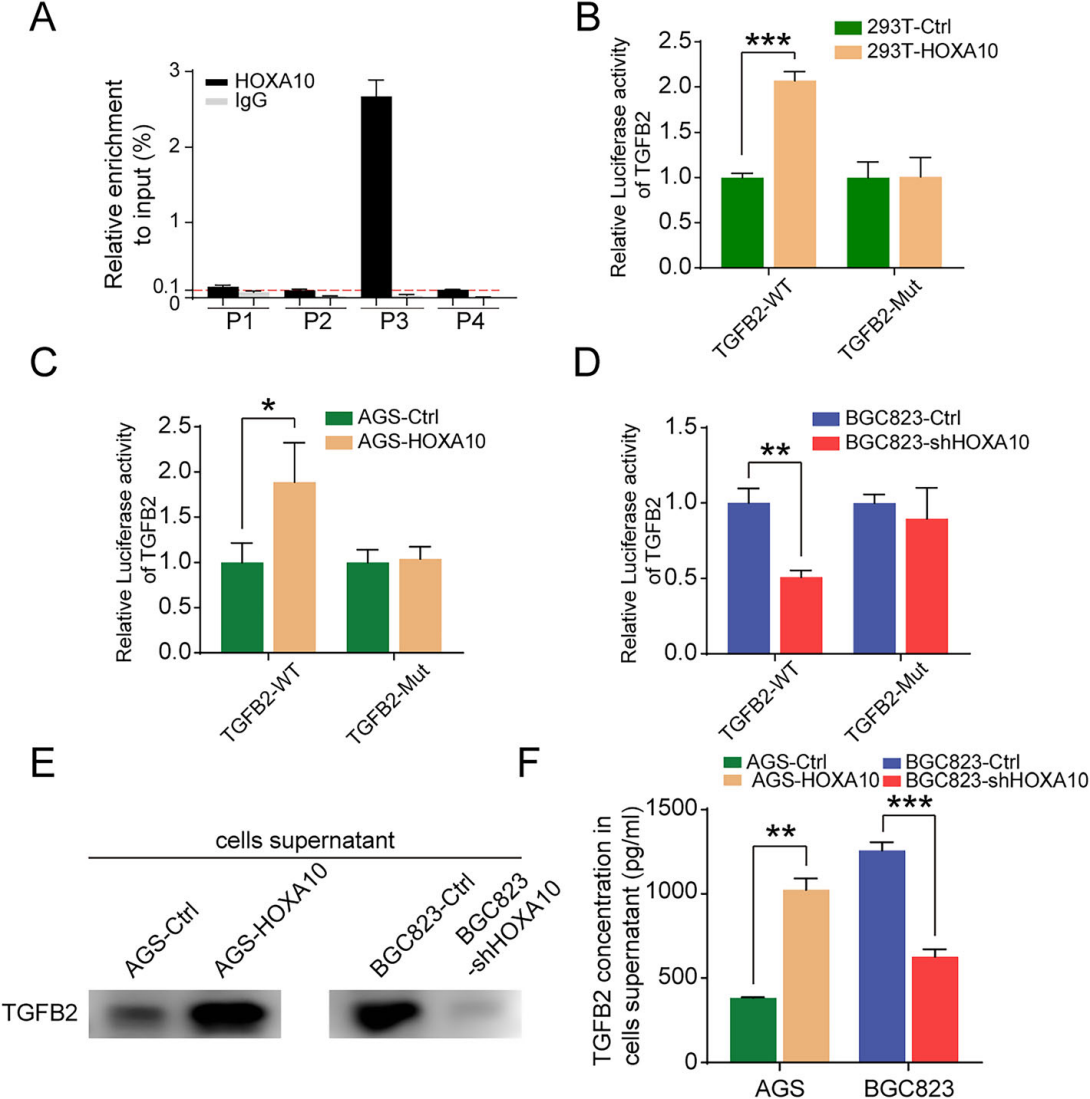
HOXA10 bound to the promoter region of TGFB2, activated its transcription and increased its secretion. **a** ChIP-qPCR assay revealed the potential binding sites of HOXA10 in the TGFB2 promoter region. **b-d** Dual-luciferase assays demonstrated that HOXA10 activated TGFB2 transcription through direct regulation. **e-f** TCA protein precipitation and ELISA assays showed that HOXA10 promoted the expression and secretion of TGFβ2 into the extracellular medium. *P < 0.05, **P < 0.01, ***P < 0.001

To further verify whether HOXA10 took part in the transcriptional regulation of TGFB2, we conducted the ChIP-qPCR analysis. According to the prediction of the database JASPAR (http://jaspar.genereg.net/), we selected four putative HOXA10 binding sites (P1-P4) in the TGFB2 promoter, designed and synthesized the corresponding primers. ChIP-qPCR results showed that HOXA10 had a high enrichment in the P3 area (Fig. 6a), which was qualitatively confirmed by 3% agarose gel electrophoresis (Fig. S3).

Subsequently, we constructed wild-type (WT) and mutant type (Mut) TGFB2 promoter-luciferase reporter plasmids and performed dual-luciferase reporter (DLR) gene assays (Fig. S3). As shown in Fig. 6b-d, HOXA10 overexpression significantly elevated TGFB2 promoter activity after the co-transfection of wild-type TGFB2. Nevertheless, luciferase activity remained unchanged when co-transfected with mutant TGFB2 plasmid.

Following up, we performed TCA precipitation and ELISA assays with cell supernatants to investigate whether HOXA10 could increase the secretion of TGFβ2 into extracellular space. The results showed that TGFβ2 content in the cell supernatants increased with HOXA10 overexpression, which indicated that HOXA10 could promote the secretion of TGFβ2 (Fig. 6e, f).

These results above confirmed TGFB2 as a target gene transcriptionally regulated by HOXA10.

### HOXA10 increases Smad2/3 expression in the cell nucleus and promotes the deposition of METTL3

Elevated phosphorylated Smad2/3 protein and increased Smad2/3 expression in the nucleus are considered a symbol of the TGFβ signaling activation.

**Fig. 7:**
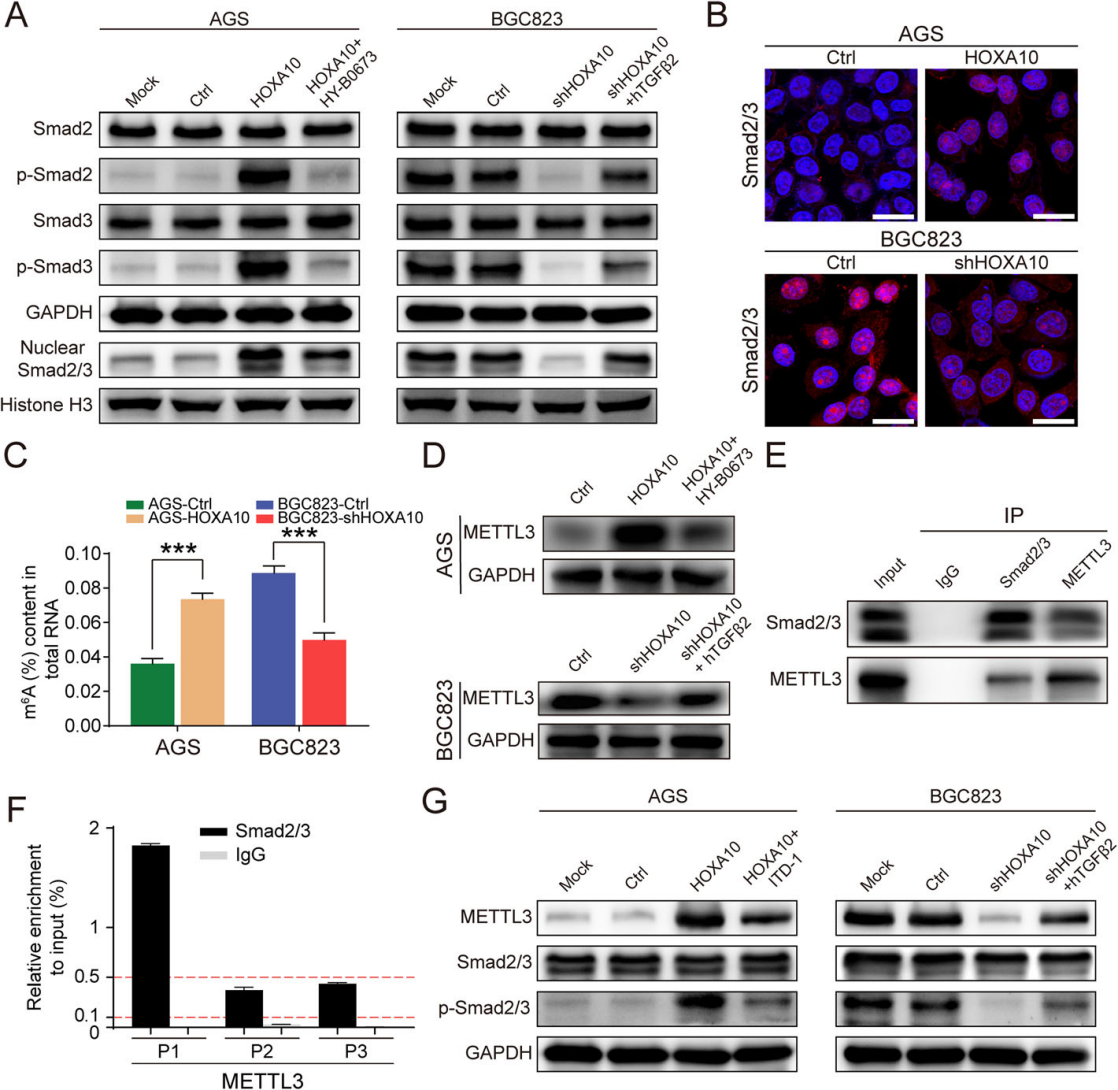
HOXA10 enhanced Smad2/3 expression in the cell nucleus and promoted the deposition of METTL3. **a** Western blot suggested that HOXA10 activated the Smad pathway but could be suppressed by TGFB2 inhibitor. **b** Confocal immunofluorescent staining indicated that HOXA10 promoted Smad2/3 translocation into the cell nucleus. **c** The colorimetric m^6^A assay demonstrated that HOXA10 elevated the m^6^A levels in GC cells. **d** Western blot showed that HOXA10 elevated METTL3 expression but could be repressed by TGFB2 inhibitor. **e** CoIP experiment revealed that Smad2/3 interacted with METTL3 in the nucleus of BGC823 cells. **f** ChIP-qPCR assay revealed the potential binding sites of Smad2/3 in the METTL3 promoter region. **g** Western blot showed that the protein expressions of P-Smad2/3 and METTL3 in AGS-HOXA10 cells were both reduced after being treated with ITD-1. Scale bars: 25 μm. ***P < 0.001

Therefore, to investigate whether HOXA10 overexpression activated the TGFβ/Smad pathway, we detected Smad2, Smad3, P-Smad2, P-Smad3, and nuclear Smad2/3 by western blot. As illustrated in Fig. 7a, increased expression of P-Smad2, P-Smad3 and nuclear Smad2/3 were detected in AGS-HOXA10 cells. Besides, confocal immunofluorescence showed that Smad2/3 markedly elevated in the nucleus with HOXA10 overexpression (Fig. 7b). These findings suggested that overexpression of HOXA10 activated the TGFβ/Smad signaling pathway in GC.

Moreover, as shown in Fig. 7a, the protein expressions of P-Smad2, P-Smad3 and nuclear Smad2/3 were reduced when AGS-HOXA10 cells were treated with TGFB2 inhibitor (HY-B0673), which indicated that HOXA10 could not activate the Smad pathway after TGFB2 depletion.

Notably, Alessandro Bertero et al. developed an optimized CoIP protocol and identified 89 putative interacting proteins of Smad2/3 in given cell types. The study focused on the relationship between Smad2/3 and the m^6^A methyltransferase complex and uncovered that Smad2/3 could interact with the METTL3-METTL14-WTAP complex [23].

Then we decided to explore whether HOXA10 was involved in m^6^A modification. The colorimetric m^6^A assay results demonstrated that the m^6^A levels were remarkably higher in AGS-HOXA10 cells, which meant HOXA10 overexpression elevated the m^6^A level in GC cells (Fig. 7c).

According to the previously reported researches, METTL3 was the main m^6^A catalytic subunit and influenced the total m^6^A methylation [32]. Therefore, we intended to evaluate whether the METTL3 expression in GC cells would vary with the change of HOXA10 expression. Western blot showed that METTL3 expression increased in AGS-HOXA10 cells but decreased in BGC823-shHOXA10 cells. Besides, we also found METTL3 expression could be inhibited by TGFB2 inhibitor or enhanced by hTGFβ2, which suggested TGFB2/Smad pathway exerted important functions in HOXA10-mediated METTL3 expression (Fig. 7d).

Subsequently, we performed CoIP and ChIP-qPCR experiments to explore the relationship between Smad2/3 and METTL3. The CoIP results demonstrated that Smad2/3 could directly interact with METTL3 in the nucleus of BGC823 cells (Fig. 7e).

Moreover, to further investigate whether Smad2/3 could also participate in the transcriptional regulation of METTL3, we selected three putative Smad2/3 binding sites (P1-P3) in the METTL3 promoter according to JASPAR and performed the ChIP experiment. ChIP-qPCR results showed that Smad2/3 had a relatively high enrichment in the P1 area of the METTL3 promoter region (Fig. 7f).

Furthermore, we used ITD-1 (Selleck, USA), which blocked P-Smad2/3 induced by TGFβ2, to investigate whether suppressing P-Smad2/3 could reduce the expression of METTL3. As shown in Fig. 7g, the protein expressions of P-Smad2/3 and METTL3 in AGS-HOXA10 cells were both inhibited after being treated with ITD-1. The above two experiments showed that Smad proteins played an important role in mediating METTL3 expression.

These results gave us valuable hints that HOXA10 might further promote METTL3 expression and elevate m^6^A level in GC cells following the activation of the TGFβ/Smad signaling pathway.

### HOXA10 regulates EMT progression partly through modulating METTL3

**Fig. 8:**
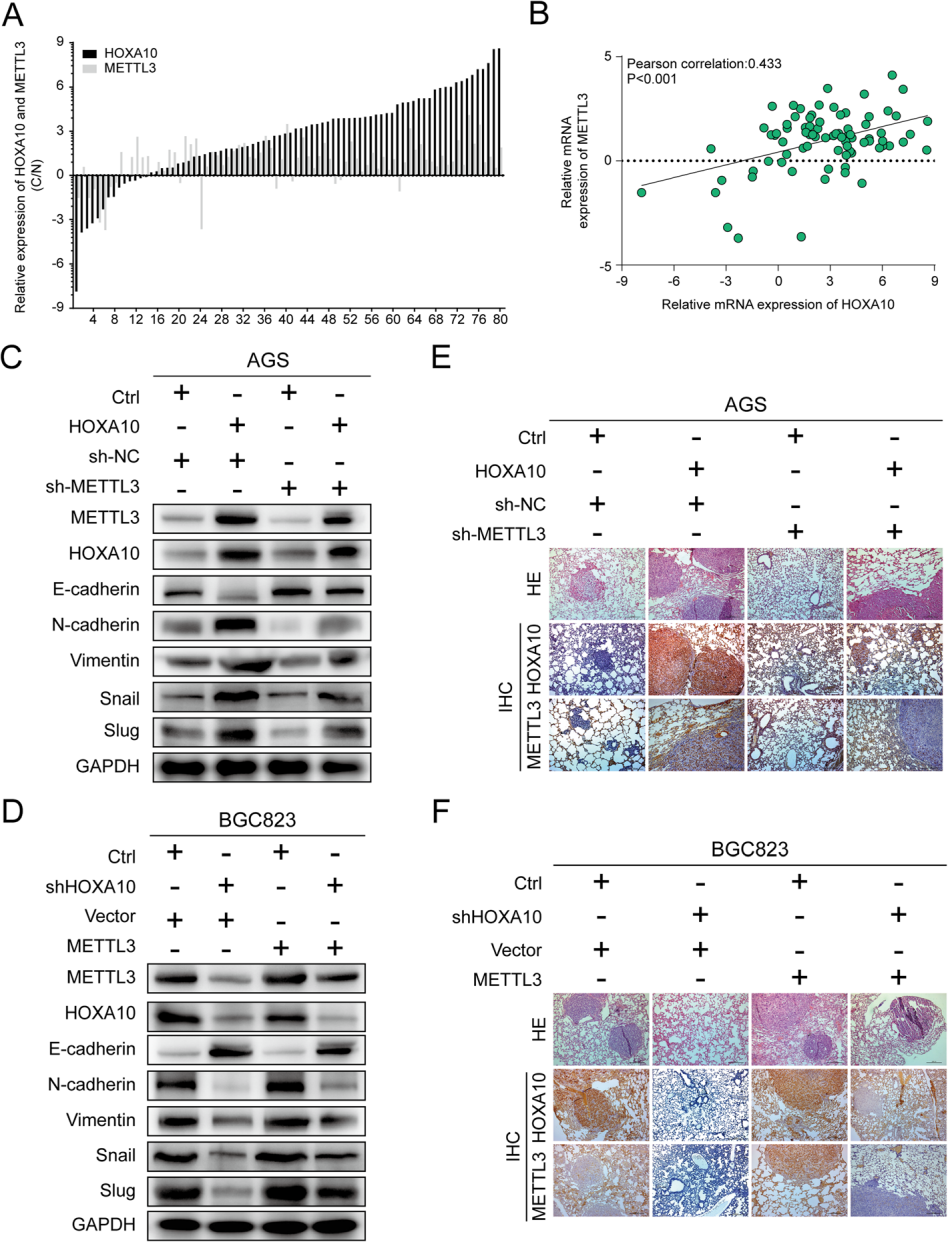
HOXA10 regulated EMT progression partly through modulating METTL3. **a-b** qRT-PCR assays and correlation analysis revealed an evident correlation between HOXA10 and METTL3 mRNA expression in 80 paired GC tissues (r = 0.433, *P* < 0.001). **c-d** Western blot showed the protein levels of EMT-related proteins in AGS-Ctrl and AGS-HOXA10 cells transfected with lentiviruses carrying sh-NC/sh-METTL3 or in BGC823-Ctrl and BGC823-shHOXA10 cells transfected with Vector/METTL3. **e-f** IHC analysis demonstrated that the lung metastasis burden caused by AGS-HOXA10 cells were alleviated with reduced staining by inhibiting METTL3. In contrast, the fewer lung colonized nodules induced by HOXA10 knockdown in BGC823-shHOXA110 cells were partly elevated with enhanced staining after overexpressing METTL3. Original magnification, 100 ×

To identify the clinical relevance of HOXA10 and METTL3, the other 80 pairs of GC samples were used for qPCR experiments. qPCR results showed HOXA10 and METTL3 mRNA expression were both upregulated in the GC samples (HOXA10, 66/80, 82.5%; METTL3, 64/80, 80%) and the Pearson correlation coefficient of r = 0.433 (*P* < 0.001) (Fig. 8a-b).

Furthermore, we determined to explore whether METTL3 could participate in the HOXA10-mediated EMT process in GC cells. Firstly, AGS-Ctrl and AGS-HOXA10 cells were transfected with lentiviruses carrying sh-METTL3 or sh-NC. Besides, BGC823-Ctrl and BGC823-shHOXA10 cells were transfected with lentiviruses carrying METTL3 or Vector. As illustrated in Fig. 8c, knockdown of METTL3 partly elevated E-cadherin levels but reduced the expression of N-cadherin, Vimentin, Snail and Slug in AGS-HOXA10 cells. In contrast, overexpression of METTL3, to a certain extent, weakened the inhibition of the EMT process caused by HOXA10 knockdown (Fig. 8d).

More importantly, we demonstrated the rescue effects of METTL3 for HOXA10-induced EMT in nude mice lung metastasis models. The increased metastatic potential induced by HOXA10 overexpression could be partially weakened by METTL3 inhibition, whereas the suppressive effect of HOXA10 knockdown on lung metastasis could be in part reversed by METTL3 upregulation (Fig. S4).

**Fig. 9:**
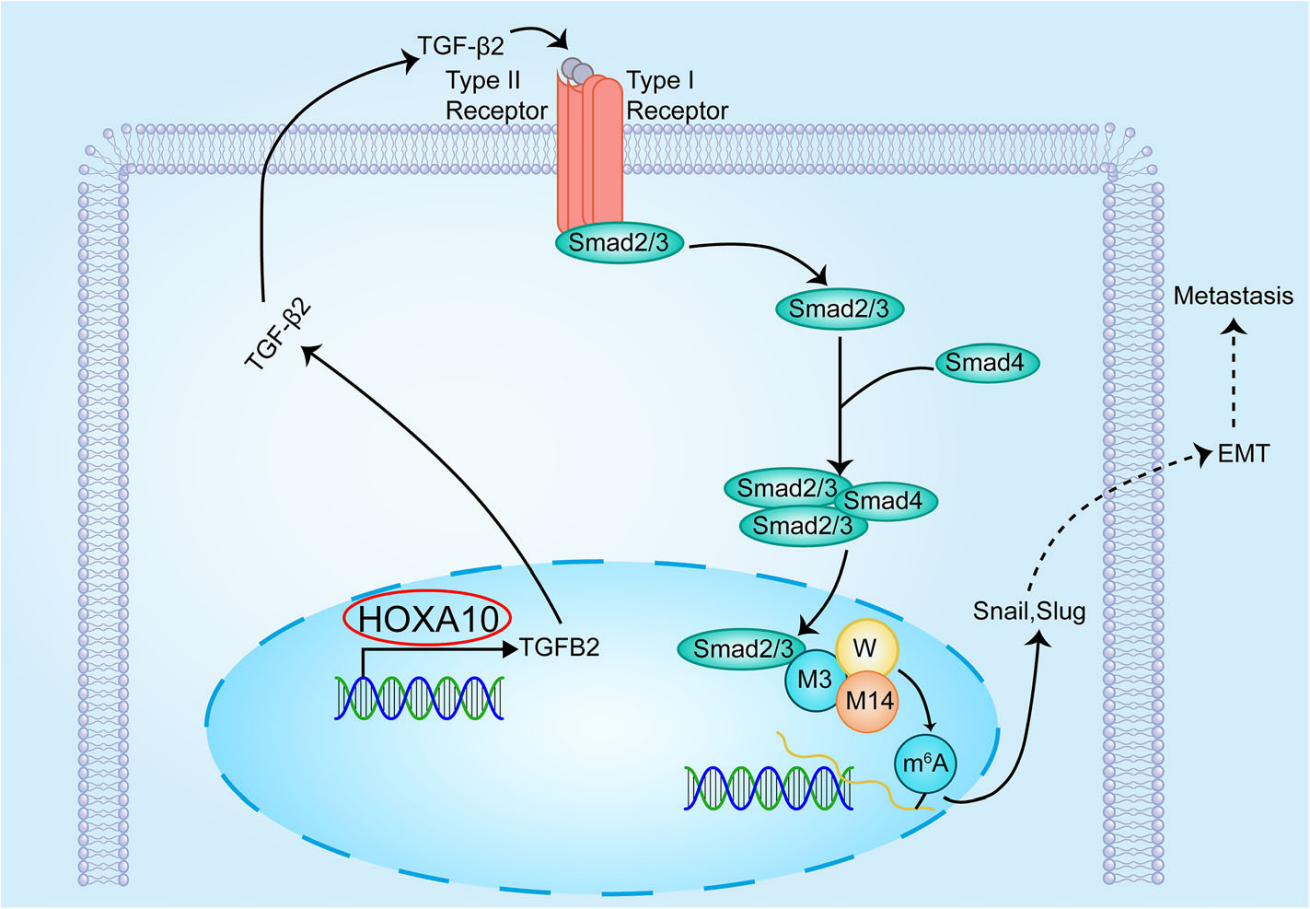
The mechanistic illustration showed HOXA10/TGFB2/Smad/METTL3 signaling axis in GC EMT progression. M3: METTL3; M14: METTL14; W: WTAP. M3/M14/W represents the METTL3-METTL14-WTAP complex

Moreover, immunohistochemistry results showed that the protein level of METTL3 was upregulated with increased lung metastasis burden or decreased with fewer lung nodules caused by altered HOXA10 expression (Fig. 8e-f). Taken together, these findings demonstrated that the upregulation of METTL3 was partly responsible for HOXA10-mediated EMT progression in GC (Fig. 9).

## Discussion

In recent years, researches concerning the relation between HOXA10 and tumor progression have been gradually increasing [35–37]. However, the underlying metastatic mechanism of HOXA10 in GC remains poorly elucidated. In the present study, we have provided experimental evidence that HOXA10 might accelerate the EMT progression in GC by activating TGFB2/Smad/METTL3 signaling, thus promoting the invasion and metastasis of GC cells.

Immunohistochemistry revealed that the expression of HOXA10 was elevated in GC, and it was noteworthy that the IS score was even higher in samples with LNM. By performing in vitro cell assays and in vivo mice metastasis models, we further observed HOXA10 upregulation significantly promoted migratory, invasive and metastatic abilities of GC cells. These findings suggested that HOXA10 might play an essential role in accelerating GC progression and metastasis.

To further elucidate the potential metastatic mechanism, RNA-Seq and GSEA were performed and the results indicated that HOXA10 knockdown inhibited the EMT pathway. EMT was thought to be a crucial contributor to promote invasion and metastasis in cancer cells. Snail (also known as SNAI1) and Slug (also known as SNAI2) are important EMT-TFs. They are both members of the zinc-fingerE-box binding homeobox factor and could repress the E-cadherin expression, which is a crucial event in triggering the EMT process [12, 13, 38].

Following the GSEA indication, cell assays showed that HOXA10 knockdown increased the E-cadherin levels but reduced the expression of Snail and Slug. Moreover, in GC tumor samples, HOXA10 was negatively correlated with E-cadherin (r = − 0.469, *P* < 0.001). These results above supported that HOXA10 promoted the EMT process in GC and triggered metastasis.

EMT progression could be activated by multiple intracellular signaling pathways, including the TGFβ, Wnt, Notch, etc. [12]. Accumulating research has recognized that TGFβ signaling plays a vital role in inducing GC EMT progression [15–17, 19].

To explore whether HOXA10 acted in promoting EMT through TGFβ signaling, we treated AGS-HOXA10 cells with TGFβ/Smad inhibitor (HY-N0439). Then we observed an elevation of E-cadherin, suggesting that HOXA10 might regulate the TGFβ pathway to promote EMT.

Three TGF-βs (TGFβ1, TGFβ2 and TGFβ3) have been identified, which could activate TGFβ signaling by interacting with specific receptors in the cell-surface [13]. Previous studies demonstrated that higher expression of TGFβs indicated poor survival in cancer patients [19, 39]. To determine which subtype of TGF-βs was the main downstream effector of HOXA10, qRT-PCR and western blot were performed. The results showed that only TGFB2 expression was significantly increased or reduced by upregulating or inhibiting HOXA10.

Then we observed that the change of cell migration or invasion abilities induced by HOXA10 were partly negatively and positively affected with TGFβ2 inhibitors and hTGFβ2 cytokine. Therefore, we further convinced that TGFB2, among three TGF-β subtypes, might be an essential effector by which HOXA10 exert functions.

To further understand the mechanism of HOXA10-regulated TGFB2 gene expression, we conducted ChIP experiments and DLR assays. ChIP-qPCR and DLR assays results indicated that HOXA10 was capable of binding to the promoter region of TGFB2 and could enhance TGFB2 transcription activity, suggesting that HOXA10 could transcriptionally upregulate TGFB2 expression.

However, in order to activate TGFβ signaling pathway, TGFβ2 needed to be secreted into the extracellular space and then bonded to the TGFβ receptor on the cell surface [40]. Consequently, we adopted TCA precipitation and ELISA experiments to detect extracellular TGFβ2 levels. The results indicated that HOXA10 upregulation could increase the secretion of TGFβ2 in extracellular supernatants.

The TGFβ signaling pathway exerted its effects through phosphorylating Smad2/3 and then translocating them into the cell nucleus [13]. As illustrated in Fig. 7a-b, the protein expression of P-Smad2, P-Smad3 and nuclear Smad2/3 elevated with HOXA10 overexpression, which suggested that HOXA10 activated the TGFβ pathway.

Notably, suppressed expression of P-Smad2, P-Smad3 and nuclear Smad2/3 were observed after AGS-HOXA10 cells were treated with TGFB2 inhibitor (HY-B0673), which indicated that TGFB2 was a key molecule for HOXA10 in activating the Smad pathway.

A recent research showed that Smad2/3 could interact with 89 potential interactomes in given cell types. Most notably, the study validated the interaction of Smad2/3 with METTL3-METTL14-WTAP complex and Smad2/3 facilitated m^6^A modification onto nascent transcripts [23].

m^6^A modification is the most common mRNA modification mainly mediated by m^6^A “writers,” “erasers,” and “readers.” It has been associated with tumor progression in glioblastoma, liver cancer, breast cancer, cervical cancer, etc. [25, 33, 41]. METTL3, METTL14 and WTAP are the main partners of “writers” belonging to m^6^A modulators, of which METTL3 is the primary catalytic subunit and has proved essential in promoting metastasis in several cancers [26–28, 32].

Most importantly, recent studies have demonstrated that METTL3 enhanced the EMT process by affecting Snail expression in HCC [32, 33]. As critical members of zinc finger transcription factors belonging to the Snail family, Snail and Slug are critical downstream effectors of Smad2/3 and they accelerate EMT by repressing E-cadherin expression [12–14, 21, 22].

Therefore, we performed the colorimetric m^6^A assay and found the m^6^A levels of AGS-HOXA10 cells were obviously higher than that in AGS-Ctrl cells. Then we investigated and observed that the changes of METTL3 expression were consistent with the altered expression of HOXA10. Besides, the upregulation of METTL3 in AGS-HOXA10 could be suppressed by HY-B0673, the TGFβ2 inhibitor, suggesting the TGFB2/Smad pathway played an important role in mediating METTL3 expression.

Afterward, we performed CoIP experiments and validated that Smad2/3 and METTL3 could be coprecipitated with each other, which was in accord with the previous results by Alessandro Bertero et al. Interestingly, we also found that Smad2/3 might bound to the promoter of METTL3 by conducting the ChIP-qPCR experiment. Moreover, after AGS-HOXA10 cells were treated with ITD-1, the expressions of METTL3 and P-Smad2/3 were both reduced. In brief, the above experiments showed that Smad proteins exerted an important effect in modulating METTL3 expression.

Subsequently, we tested the correlation between HOXA10 and METTL3 in other 80 paired GC samples and the results showed evident correlation (r = 0.433, *P* < 0.001). More importantly, western blot showed that the expressions of important EMT-TFs, Snail and Slug, were reduced, and E-cadherin was upregulated when METTL3 was inhibited in AGS-HOXA10 cells. Furthermore, the in vivo lung metastatic models demonstrated that METTL3 knockdown weakened the metastatic potentials induced by HOXA10. These experiments suggested that METTL3 was an important molecule involved in HOXA10-mediated EMT.

## Conclusion

In summary, our study preliminarily revealed that elevated HOXA10 expression in GC was significantly correlated with LNM. Overexpression of HOXA10 played a pivotal role in promoting EMT and GC metastasis by modulating TGFB2/Smad/METTL3 signaling axis, thus might help provide a novel candidate for GC treatment and develop new therapeutic strategies for GC metastasis.

## Supplementary Information

**Additional file 1: Figure S1.** GSEA comparing BGC823-Ctrl cells with BGC823-shHOXA10 cells: the level of HOXA10 mRNA was positively correlated with Hallmark Epithelial-Mesenchymal Transition.

**Additional file 2: Figure S2.** The statistical graph of the migration and invasion cells in Fig. 5d and e. **P < 0.01, ***P < 0.001.

**Additional file 3: Figure S3.** Qualitative analysis of ChIP-qPCR results with 3% agarose gel electrophoresis and a schematic diagram of the TGFB2-luciferase mutation sites.

**Additional file 4: Figure S4.** In vivo metastasis models of AGS or BGC823 cells by suppressing or elevating METTL3 expression, respectively. **P < 0.01, ***P < 0.001.

## Data Availability

All data generated and analyzed in this study are included in this article and its supplementary information files.

## Abbreviations

GC: Gastric cancer
IHC: Immunohistochemistry
TGFβ: Transforming growth factor beta
GSEA: Gene Set Enrichment Analysis
EMT: Epithelial-mesenchymal transition
DLR: Dual-luciferase reporter
CoIP: Co-immunoprecipitation
ChIP: Chromatin immunoprecipitation
LNM: Lymph node metastasis
m^6^A: N6-methyladenosine
PBS: Phosphate-buffered saline
FBS: Fetal bovine serum
