# Supplementary figures and images for: HOXA10 mediates epithelial-mesenchymal transition to promote gastric cancer metastasis partly via modulation of TGFB2/Smad/METTL3 signaling axis
Source: J Exp Clin Cancer Res. 2021 Feb 9;40:62. doi: 10.1186/s13046-021-01859-0 (PMC7874610; doi:10.1186/s13046-021-01859-0)

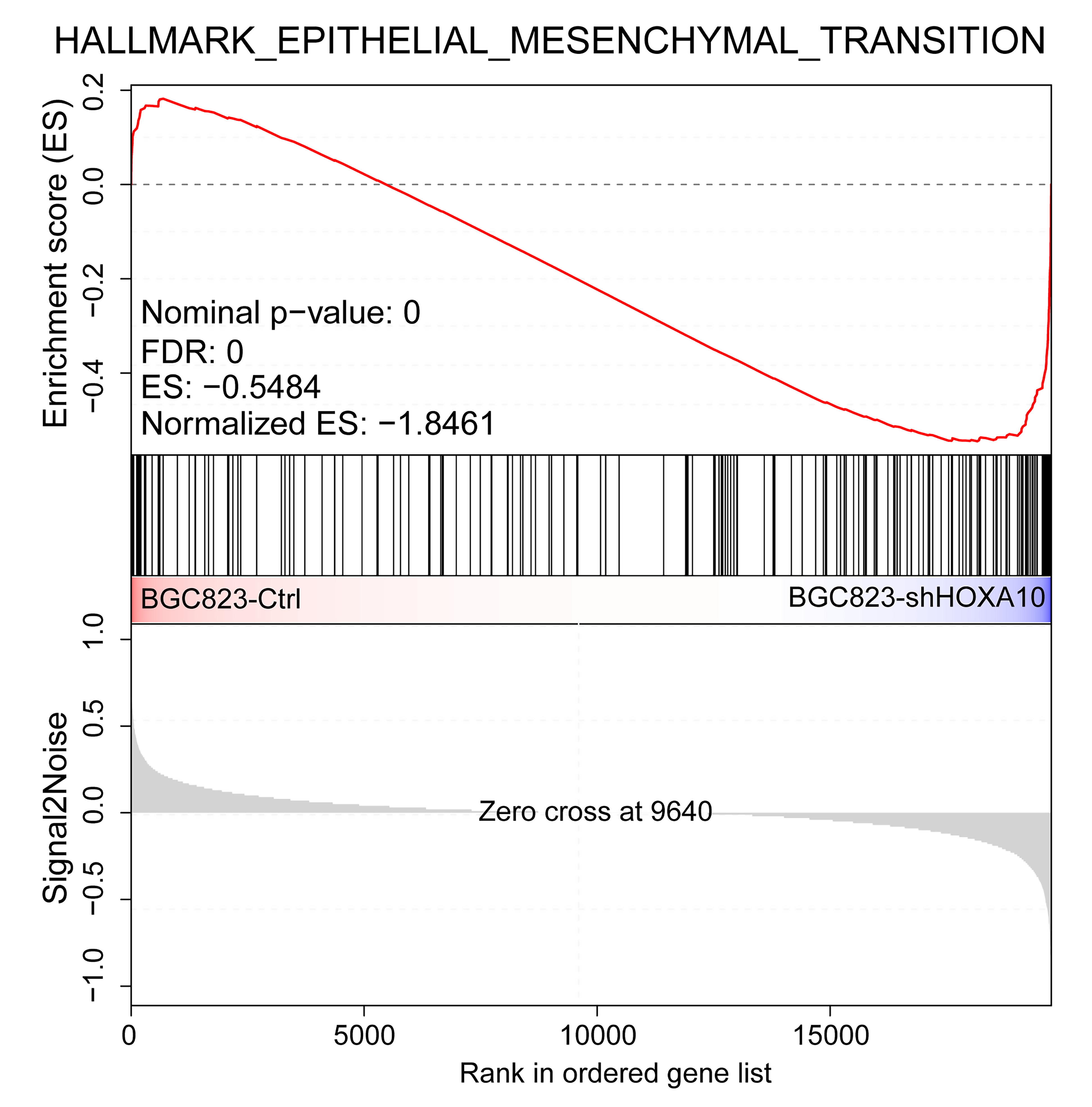

Supplement: Supplementary file 1 — Additional file 1: Figure S1. GSEA comparing BGC823-Ctrl cells with BGC823-shHOXA10 cells: the level of HOXA10 mRNA was positively correlated with Hallmark Epithelial-Mesenchymal Transition. [file 13046_2021_1859_MOESM1_ESM.tif]

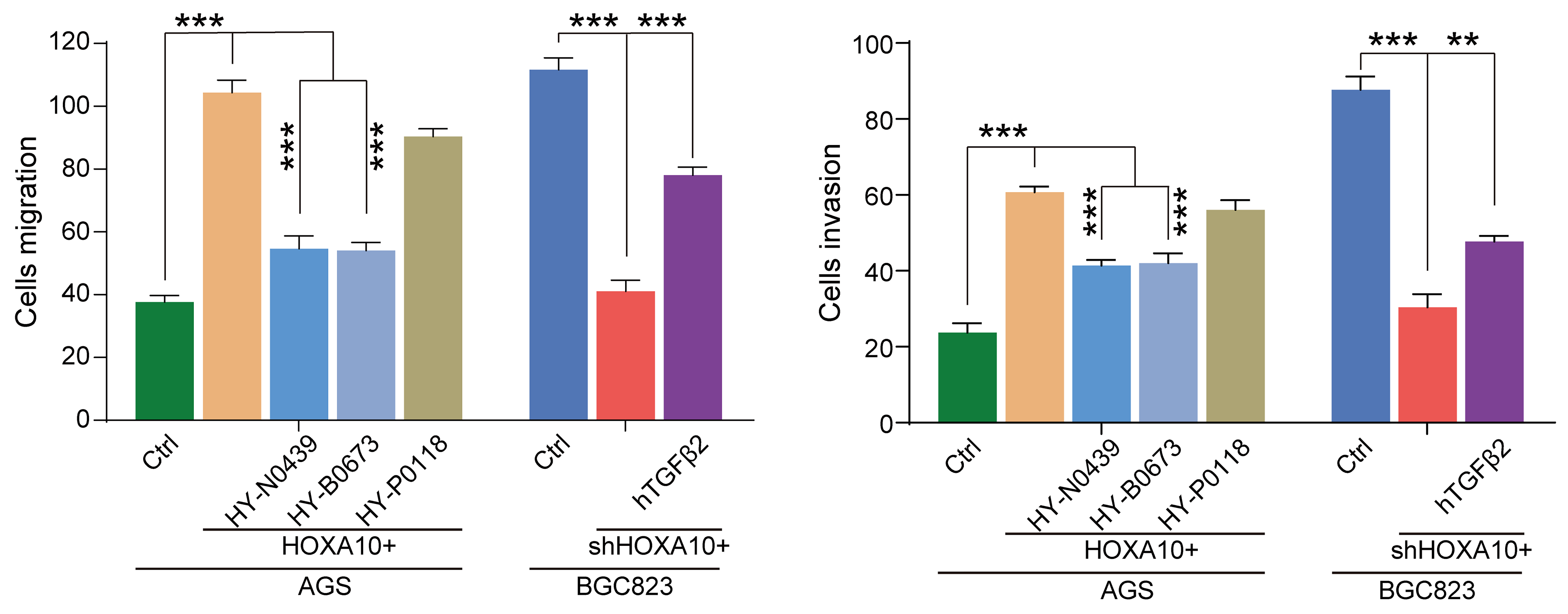

Supplement: Supplementary file 2 — Additional file 2: Figure S2. The statistical graph of the migration and invasion cells in Fig. 5d and e. **P < 0.01, ***P < 0.001. [file 13046_2021_1859_MOESM2_ESM.tif]

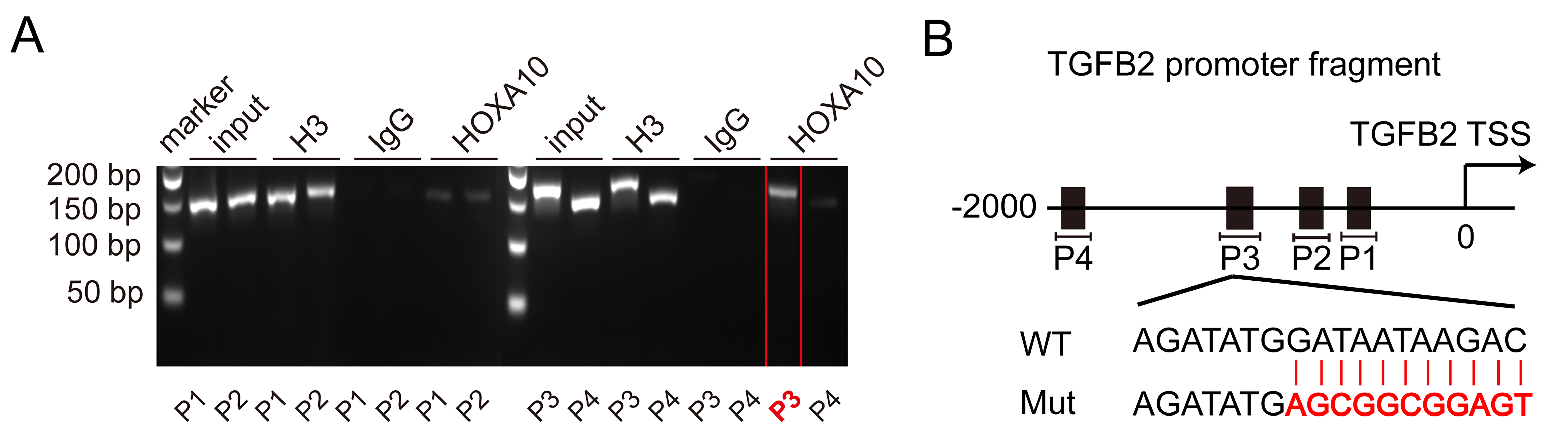

Supplement: Supplementary file 3 — Additional file 3: Figure S3. Qualitative analysis of ChIP-qPCR results with 3% agarose gel electrophoresis and a schematic diagram of the TGFB2-luciferase mutation sites. [file 13046_2021_1859_MOESM3_ESM.tif]

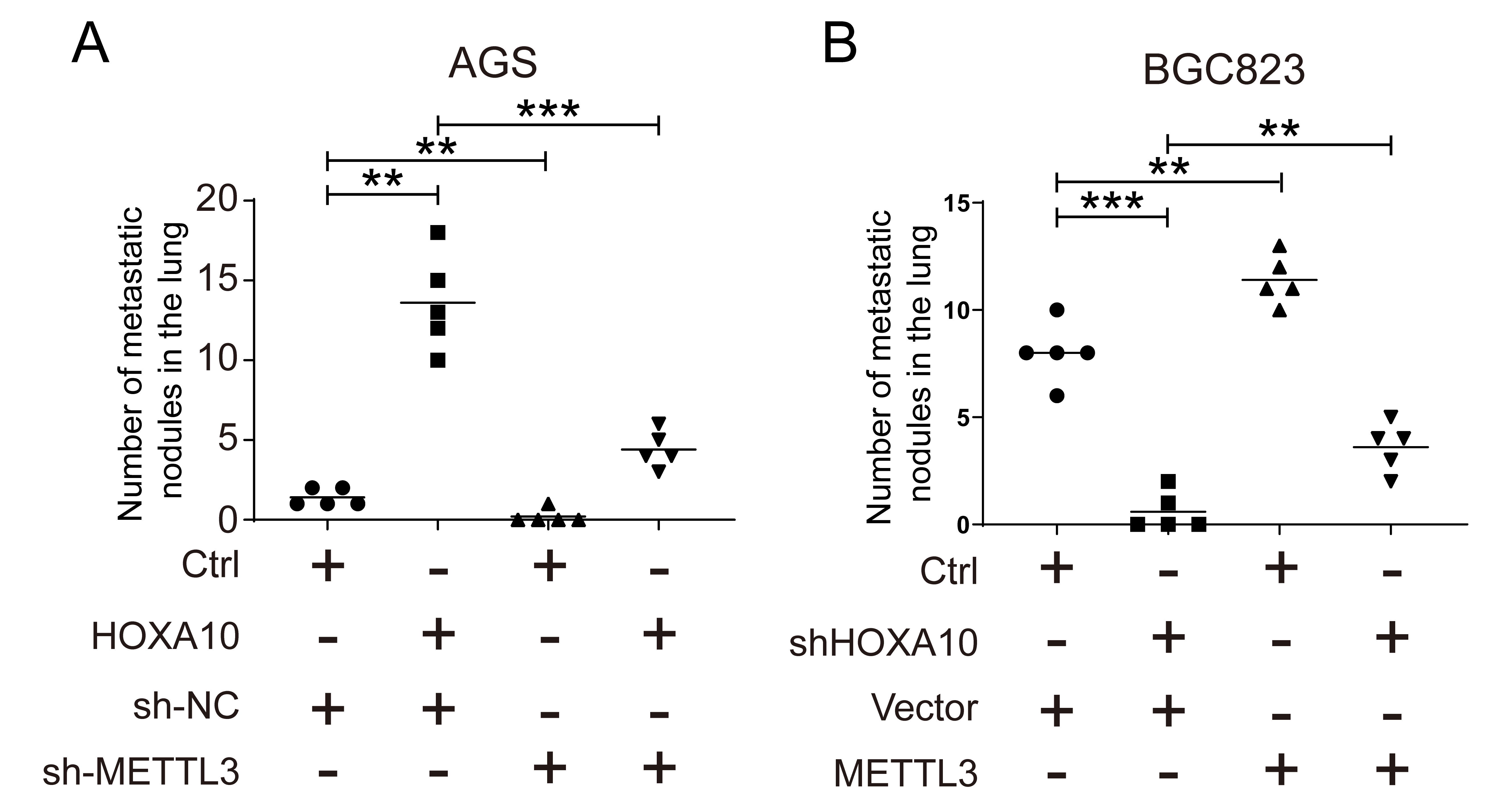

Supplement: Supplementary file 4 — Additional file 4: Figure S4. In vivo metastasis models of AGS or BGC823 cells by suppressing or elevating METTL3 expression, respectively. **P < 0.01, ***P < 0.001. [file 13046_2021_1859_MOESM4_ESM.tif]
